# Inhibitory effect of NBL1 on PDGF-BB-induced human PASMC proliferation through blockade of PDGFβ-p38MAPK pathway

**DOI:** 10.1042/BSR20160199

**Published:** 2016-08-31

**Authors:** Chuanjue Cui, Hongliang Zhang, Lin-Na Guo, Xiaoling Zhang, Liukun Meng, Xiangbin Pan, Yingjie Wei

**Affiliations:** * State Key Laboratory of Cardiovascular Disease, Fuwai Hospital, National Center for Cardiovascular Diseases, Chinese Academy of Medical Sciences and Peking Union Medical College, Beijing 100037, People's Republic of China; † Department of Cardiology, First Affiliated Hospital of Jiamusi University, Jiamusi, Heilongjiang 154002, People's Republic of China

**Keywords:** human pulmonary artery smooth muscle cells, neuroblastoma suppressor of tumorigenicity 1, platelet-derived growth factor-BB (PDGF-BB), p38 mitogen-activated protein kinase (p38MAPK), proliferation

## Abstract

Pulmonary artery remodelling is a key feature in the pathological progress of pulmonary arterial hypertension (PAH). Moreover, excessive proliferation of pulmonary arterial smooth muscle cells (PASMCs) plays a critical role in the pathogenesis of pulmonary artery remodelling. Neuroblastoma suppressor of tumorigenicity 1 (NBL1) has been previously shown to induce growth inhibition in tumour cells. However, the effect of NBL1 in the regulation of human PASMC proliferation remains unclear. In cultured human PASMCs, we observed a dose-dependent inhibitory effect of NBL1 on platelet derived growth factor (PDGF)-BB-induced cell growth, DNA synthesis and proliferating cell nuclear antigen (PCNA) expression, as measured by MTS assay, 5-ethynil-2-deoxyuridine (EdU) analysis and western blots respectively. We also detected the expression and activities of cell-cycle positive regulators (cyclin D1, cyclin E, CDK2, CDK4 and CDK6) and negative regulators (p21 and p27) in human PASMCs by western blots and co-immuoprecipitation (IP). Our results show that NBL1-induced growth suppression is associated with the decreased activity of cyclin D1–CDK4 and the decreased phosphorylation of p27 in PDGF-BB-treated human PASMCs. By western blots using the phosphor-specific antibodies, we further demonstrated that NBL1 induced growth suppression is mediated by blockade of the up-stream PDGF-receptor β (PDGFRβ)-p38 mitogen-activated protein kinase (MAPK). In conclusion, our results suggest that NBL1 could inhibit PDGF-BB-induced human PASMC proliferation, and the underlying mechanism is associated with the decreased cyclin D1–CDK4 activity and up-regulated p27 by decreasing the phosphorylation of p27 via blockade of PDGFRβ-p38MAPK signal cascade. Our findings may provide a potential therapeutic target for PAH.

## INTRODUCTION

Pulmonary arterial hypertension (PAH) is a progressive disorder with high morbidity and mortality. Pulmonary artery remodelling is a key feature in the pathological progress of PAH. Moreover, excessive proliferation of pulmonary arterial smooth muscle cells (PASMCs) is critical in the pathogenesis of pulmonary artery remodelling [1]. Therefore, it will be helpful to find an effective molecular of anti-proliferation of PASMCs for therapeutic purpose.

Platelet-derived growth factor (PDGF) is one of the numerous growth factors that regulate cell growth and division. PDGF is a potent mitogen for cells of mesenchymal origin, including fibroblasts, smooth muscle cells and glial cells. In chemical terms, PDGF is a dimeric glycoprotein composed of two A (-AA) or two B (-BB) chains [2]. PDGF-BB induces the proliferation and migration of PASMCs and has been proposed to be a key mediator in the progression of PAH [3]. Thus the inhibition of PDGF-BB stimulated PASMCs proliferation may represent an important target for therapeutic intervention in PAH.

Neuroblastoma suppressor of tumorigenicity 1 (NBL1) is a secreted protein which belongs the differential screening-selected gene aberrative in neuroblastoma (DAN) subfamily [4]. It was initially identified as a gene whose expression is suppressed in v-src-transformed rat fibroblast 3Y1 cells (SR-3Y1) [5]. Moreover, overexpression of NBL1 inhibited the proliferation of 3Y1 cells [5] and SAOS-2 cells [6]. The analysis of NBL1 function by transfection of cultured cell revealed that NBL1 may suppress the transformed phenotype and delay entry into the S-phase in tumour cells, suggesting that NBL1 has a tumour-suppressive activity [7]. Our earlier results showed a significant increase in NBL1 expression in the normal lungs, but a decrease in the lungs suffering from systemic-to-pulmonary shunts in a rat model of PAH [8]. Therefore, NBL1 might be involved in the progression of PAH.

The aim of the present study was designed to investigate whether NBL1 had an inhibitory effect on PDGF-BB-induced human PASMC proliferation, and clarify the underlying molecular mechanism.

## MATERIALS AND METHODS

### Cell culture

The human pulmonary artery smooth muscle cells (PASMCs) were purchased from ScienCell Research Laboratories and cultured in smooth muscle cell medium (SMCM, ScienCell Research Laboratories) supplemented with 2% FBS (ScienCell Research Laboratories) 1% penicillin/streptomycin (P/S) and 1% smooth muscle cell growth supplement (SMCGS, ScienCell Research Laboratories). The cells were cultured at 37°C in a 5% CO_2_ incubator, and the cell culture medium was changed every 2 days. The human PASMCs were used at passages 3–9 in experiments.

### Cell treatments

Cells were serum-starved for 24 h, then replaced with media with 0.1% FBS and the cells were incubated for additional time as indicated. The cells were pretreated with Recombinant Human NBL1 (Sino Biological) for 1 h prior to the addition of PDGF-BB (Sigma–Aldrich).

### MTS assay

To determine the effects of NBL1 on the proliferative capacity of human PASMCs, CellTiter 96 Aqueous One Solution Cell Proliferation Assay (MTS) (Promega) was used according to manufacturer's instruction with minor modification. Briefly, after treated with cells, 50 μl of CellTiter 96 Aqueous One Solution reagent was added into each well of the 96-well assay plates containing cells in 150 μl of culture medium. After 2 h incubation at 37°C and 5% CO_2_, and then absorbance measurement at 490 nm using infinite M200PRO (Tecan) microplate reader.

### EdU incorporation assay

The cell proliferation rate was determined by the uptake of 5-ethynil-2-deoxyuridine (EdU) into DNA, using a Click-iT EdU microplate assay kit (Life Technologies) according to the manufacturer's instruction manual. The incorporated EdU in DNA was coupled with Oregon Green-azide, and subsequently incubated with horseradish peroxidase (HRP)-labelled anti-Oregon Green antibody and Amplex UltraRed. The fluorescence (expressed as relative fluorescence units [RFU]) at 490 nm excitation/585 nm emission was measured with infinite M200PRO microplate reader (Tecan).

### Western blots

Cultured cells were lysed with cell lysis buffer (Beyotime). Protein samples were separated by precast NuPAGE Novex 4–12% (w/v) Bis–Tris gels (Life Technologies) and then transferred on to nitrocellulose membrane using the iBlot™ dry blotting system as described by the manufacturer (Life Technologies). Membranes were stained with Ponceau S and blocked in TBST buffer (20 mM Tris, pH 7.5, 150 mM NaCl, 0.1% tween 20) containing 5% non-fat dry milk for 1 h at room temperature. The blots were reacted with primary antibodies (anti-PCNA, anti-p-p27, anti-p27, anti-p21, anti-p-ERK, anti-ERK, anti-p-p38MAPK, anti-p38MAPK, anti-p-JNK, anti-JNK, anti-p-PDGFRβ, anti-PDGFRβ, anti-cyclin D1, anti-cyclin E, anti-CDK2, 6, Cell Signaling Technology; anti-CDK4 and anti-GAPDH, abcam) overnight at 4°C, then with a secondary antibody conjugated with HRP (Cell Signaling Technology) for 2 h at room temperature. Blots were developed using chemiluminescence (ECL, Thermo Fisher Scientific) on Fluor Chem M image system (Protein simple).

### siRNA transfection

The siRNA duplexes targeting human p27 Kip1 was purchased from Cell Signaling Technology. The RNAi negative control Duplex (Life Technologies) were used as a control. RNAi were transfected into cells using Lipofectamine™ RNAiMAX (Life Technologies) according to manufacturer's protocol.

### Co-immuoprecipitation

Human PASMCs were harvested after PDGF-BB treatment for 3 h with or without NBL1 pretreatment. Cell lysates (500 μg) were prepared for co-immuoprecipitation (co-IP) with the Catch and Release immuoprecipitation system (Millipore). An equal amount of protein was incubated with mouse monoclonal cyclin D1 antibody (Cell Signaling Technology), mouse monoclonal CDK4 antibody (abcam) and antibody capture affinity ligand at room temperature and then transferred on to spin columns. After centrifugation and washing, IP elution buffer was added on to the spin columns and elute the immune complexes. An equal volume of eluate was further analysed by western blot as described above.

### Statistical analysis

Statistical significance was determined using the Student's *t* test. Results are presented as the mean ± S.E.M. All *P*-values are two-tailed and significance was accepted when *P*<0.05.

## RESULTS

### NBL1 inhibits PDGF-BB-induced proliferation of human PASMCs

**Figure 1:**
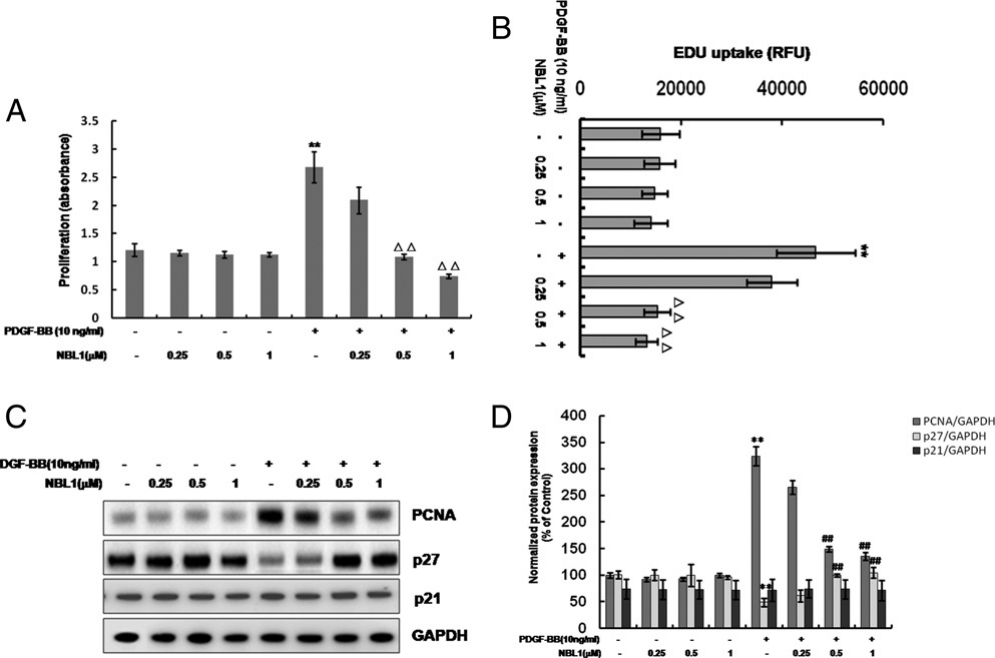
NBL1 inhibits proliferation of human PASMCs induced by PDGF-BB Human PASMCs were pretreated by various concentrations (0.25, 0.5, 1 μM) of NBL1 for 1 h and then treated with or without PDGF-BB for 24 h. (**A**) MTS proliferation assay. Data were collected with an absorbance wavelength of 490 nm. (**B**) EdU uptake analysis. After treatment, cell proliferation was determined with uptake of EdU into DNA using a Click-iT EdU microplate assay kit. The resulting fluorescence (measured in RFU) was measured. (**C**) Western blotting and densitometric analysis. (**D**) GAPDH was used as the loading protein. Data represent as mean ± S.E.M. (*N*=3). (A–B) **P<0.01, compared with control, ^△△^*P*<0.01, compared with PDGF-BB-treated cells. (D) **P<0.01 compared with control, ^##^*P*<0.01, compared with PDGF-BB treatment.

MTS assay was performed to investigate the effect of different concentrations (0.25, 0.5 and 1 μM) of NBL1 on PDGF-BB-induced proliferation of human PASMCs. The results indicated that the cell growth of human PASMCs was markedly increased following stimulation with 10 ng/ml of PDGF-BB for 24 h compared with control group. NBL1 doses between 0.5 and 1 μM were showed to inhibit the PDGF-BB-induced proliferation of human PASMCs (Figure 1A). Moreover, we determined the effect of NBL1 on the rate of cellular DNA synthesis under the same doses by EdU uptake analysis. As shown in Figure 1(B), PDGF-BB increased the EdU uptake value but NBL1 (0.5 and 1 μM) significantly blocked the EdU uptake value induced by PDGF-BB. In addition, we further examined the expression of proliferating cell nuclear antigen (PCNA) and cell cycle inhibitors, such as p21 and p27 expression. The western blots data showed that PDGF-BB increased the expression of PCNA but decreased the expression of p27. On the contrary, inhibition of cell proliferation by NBL1 was verified by its ability to decrease PCNA protein level but to increase p27 expression in a dose-dependent manner at 24 h. However, the protein level of p21 did not significantly change after treatment of PDGF-BB with or without NBL1 (Figures 1C and 1D). These results suggest that 0.5 μM of NBL1 is the lowest effective dose, thus this dose was selected for further experiments.

### NBL1 inhibits cyclin D1–CDK4 activation and phosphorylation of p27 in human PASMCs

**Figure 2:**
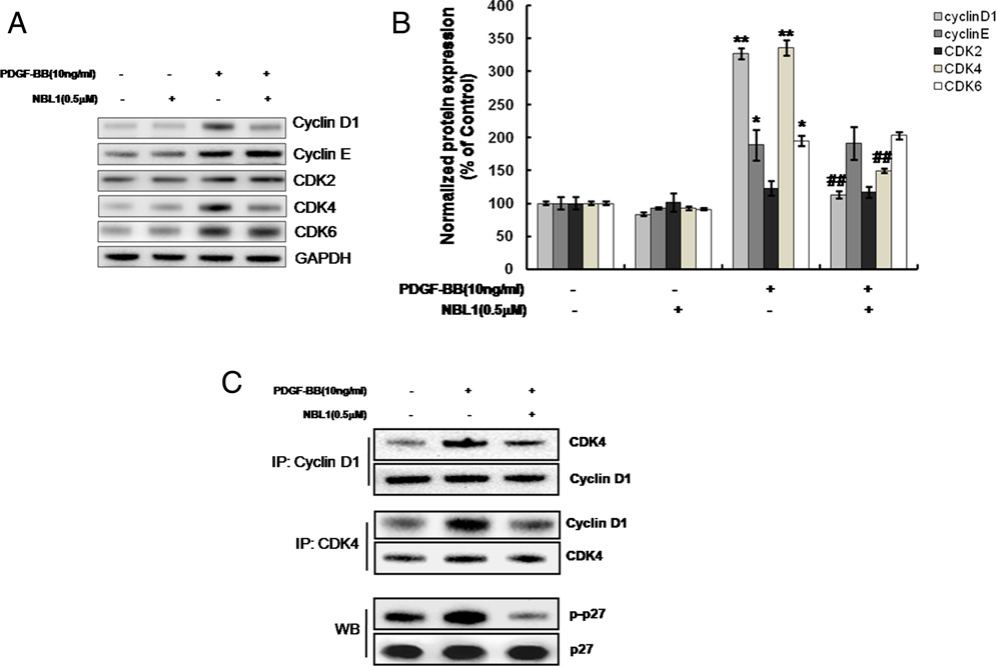
NBL1 inhibits expression and activation of cyclin D1–CDK4 and phosphorylation of p27 in human PASMCs (**A**) Western blotting and densitometric analysis. (**B**) Human PASMCs were pretreated by NBL1 (0.5 μM) for 1 h and then stimulated with PDGF-BB for 24 h. GAPDH was used as the loading protein. (**C**) Cross co-immuoprecipitation (IP) and western blots (WB). Human PASMCs were stimulated with PDGF-BB for 3 h following NBL1 pretreatment. *P<0.05, **P<0.01, compared with control, ^##^*P*<0.01, compared with PDGF-BB treatment.

**Figure 3:**
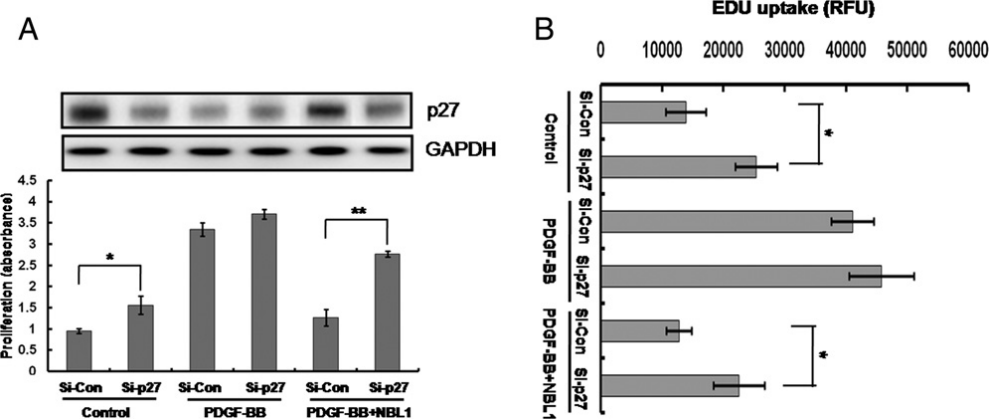
Up-regulation of p27 is involved in NBL1-induced growth suppression Human PASMCs were transfected with p27 siRNA for 24 h, and then treated with PDGF-BB for another 24 h with or without NBL1 pretreatment. (**A**) Western blots (upper) and MTS assay (below). Data were collected with an absorbance wavelength of 490 nm. (**B**) EdU uptake analysis. The resulting fluorescence (measured in RFU) was measured. Data represent as mean ± S.E.M. (*N*=3). *P<0.05, **P<0.01 compared with si-control.

G_1_- to S-phase cell cycle progression has been implicated in the formation of vascular lesions in vascular disease [9]. To investigate the regulation of cell cycle events in NBL1 inhibition, we examined the expression of G_1_- to S-phase cell cycle associated proteins including cyclin D1, cyclin E, CDK2, CDK4 and CDK6 analysing by western blotting. The results showed that the protein expressions of cyclin D1 and CDK4 but not cyclin E, CDK2 and CDK6 are significantly reduced by NBL1 (Figures 2A and 2B). We further performed co-IP using anti-cyclin D1 or anti-CDK4 antibodies to determine the activity of cyclin D1 and CDK4 complex, the co-IP results showed that cyclin D1–CDK4 complex formation induced by PDGF-BB was reduced by pretreatment of NBL1 in human PASMCs at 3 h (Figure 2C). Cyclins and CDKs regulate phosphorylation of several substrates, including p27 [10]. This phosphorylation is a prerequisite for the proteasome dependent degradation of p27 [11]. Therefore, we also detected the phosphorylation of p27 using an anti-phospho-p27 antibody (Sigma–Aldrich) under the same condition (at 3 h). The western blots results showed that the phosphorylated p27 was reduced by NBL1, which linked to the down-regulation of cyclin D1–CDK4 complex (Figure 2C). Thus, NBL1 may increase p27 protein stability by inhibiting its phosphorylation. To test the involvement of p27 in NBL1-induced growth suppression, we examined the effects of p27 knockdown by siRNA. The MTS and EdU uptake assay results showed that p27 knockdown could block the growth arrest induced by NBL1 (Figures 3A and 3B).

Taken together, NBL1 inhibits cyclin D1–CDK4 activation and promotes p27 protein expression by decreasing the phosphorylation of p27 in PDGF-BB-treated human PASMCs.

### NBL1 inhibits PDGFRβ-p38MAPK signalling cascade activated by PDGF-BB in human PASMCs

**Figure 4:**
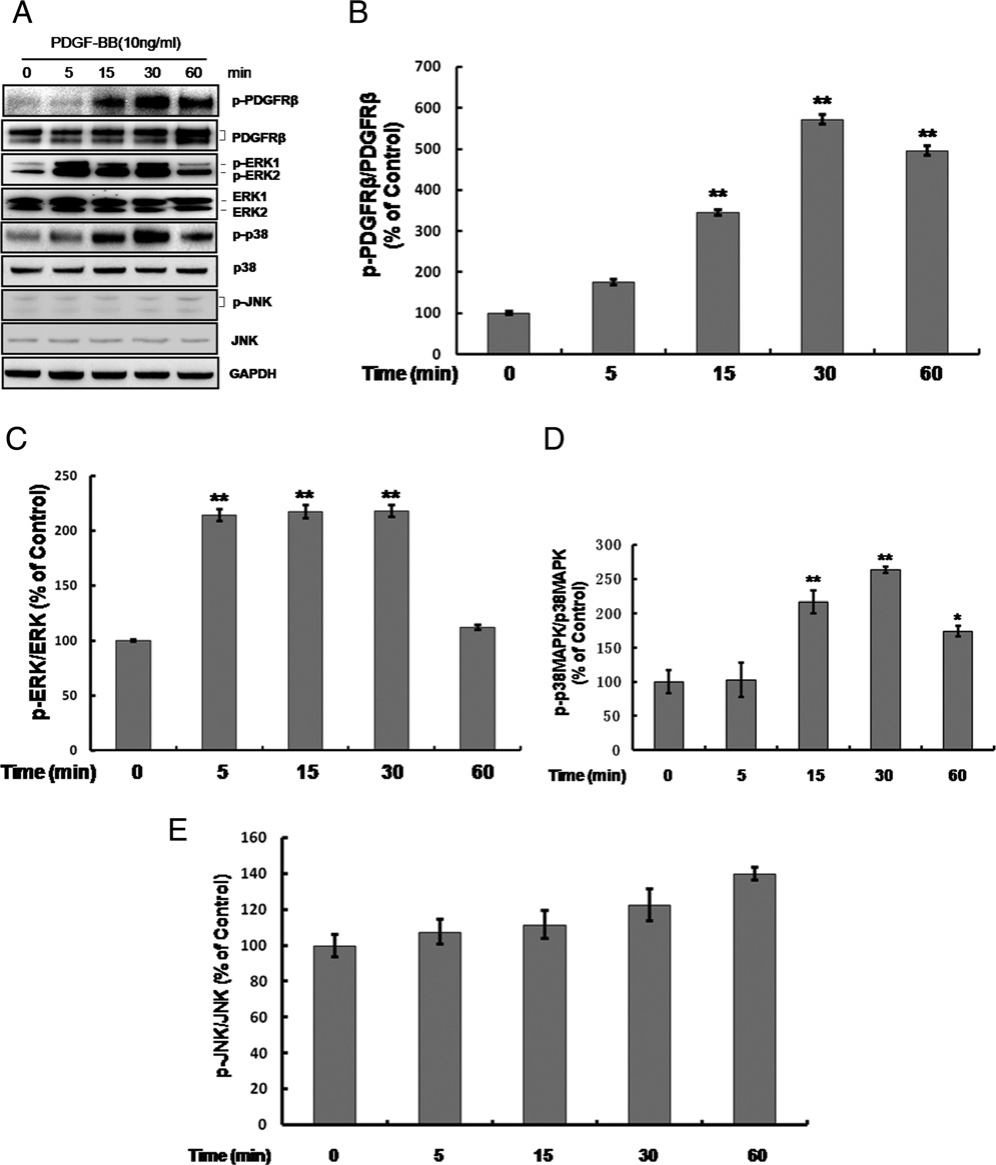
Signalling cascade activated by PDGF-BB in human PASMCs Human PASMCs were exposed to 10 ng/ml of PDGF-BB for different times as indicated. (**A**) Time course of PDGFRβ/ MAPK signalling by western blots with antibodies specific to phosphorylated PDGFRβ (p-PDGFRβ) and PDGFRβ, phosphorylated ERK1/2 (p-ERK1/2) and ERK1/2, phosphorylated p38MAPK (p-p38MAPK) and p38MAPK, phosphorylated JNK (p-JNK) and JNK. (**B**–**E**) Summarized results of the densitometric analyses of p-PDGFRβ (B), p-ERK1/2 (C), p-p38MAPK (D) and p-JNK (E). GAPDH was used as the loading protein. Data represent as mean ± S.E.M. (*N*=3). *P<0.05, **P<0.01, compared with control, ^##^*P*<0.01, compared with PDGF-BB treatment.

**Figure 5:**
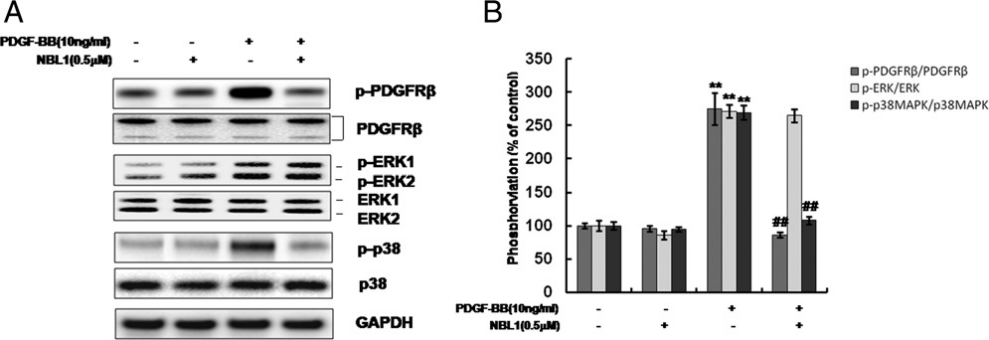
NBL1 inhibits PDGFRβ-p38MAPK signalling cascade activated by PDGF-BB in human PASMCs Treated the human PASMCs with PDGF-BB for 30 min and following pretreatment with NBL1 by western blotting (**A**) and densitometric analysis in (**B**). GAPDH was used as the loading protein. Data represent as mean ± S.E.M. (*N*=3). *P<0.05, **P<0.01, compared with control, ^##^*P*<0.01, compared with PDGF-BB treatment.

**Figure 6:**
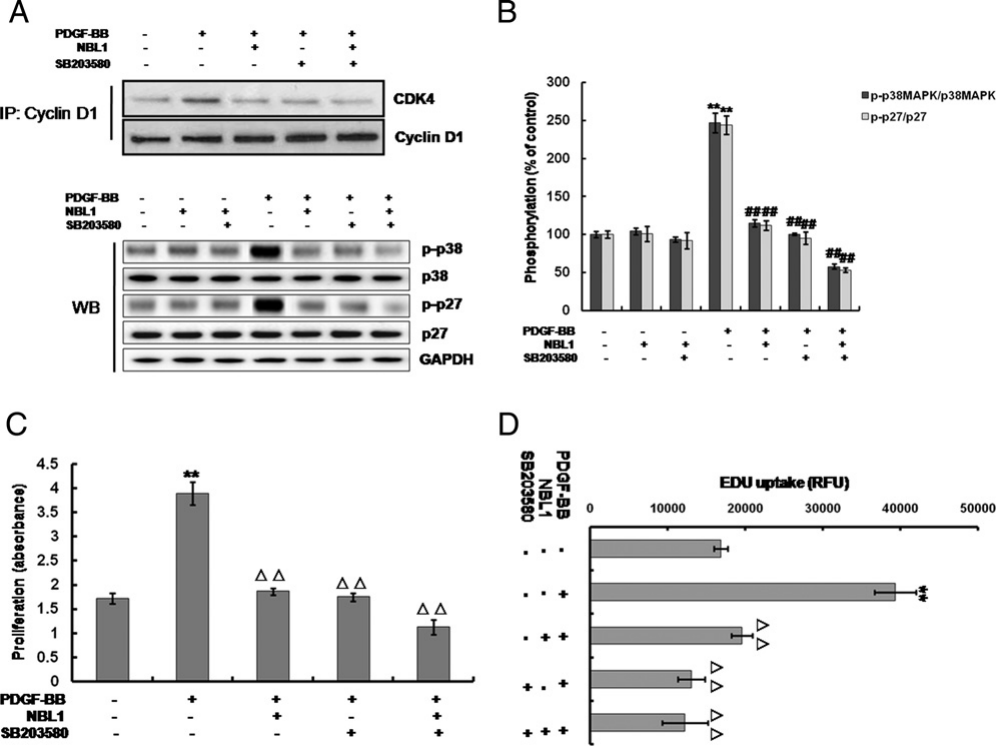
Effects of the p38MAPK inhibitor SB203580 treatment on the p27 phosphorylated level and human PASMCs growth Treated the human PASMCs with PDGF-BB for 3 h and following pretreatment with NBL1 and/or SB203580. (**A**) Western blots analysis using phosphor-specific antibodies and co-IP assay (cyclin D1–CDK4 activity). (**B**) Densitometric analysis of western blotting as shown in (A) lower. GAPDH was used as the loading protein. Treated the human PASMCs with PDGF-BB for 24 h and following pretreatment with NBL1 and/or SB203580. (**C**) MTS assay (cell proliferation). (**D**) EdU uptake analysis (cellular DNA synthesis). Data represent as mean ± S.E.M. (*N*=3). (A and B) *P<0.05, **P<0.01, compared with control, ^##^*P*<0.01, compared with PDGF-BB treatment. (C and D) **P<0.01, compared with control, ^△△^*P*<0.01, compared with PDGF-BB-treated cells.

To further clarify the possible signalling pathways underlying the inhibition of human PASMCs proliferation induced by NBL1, we estimated the effect of NBL1 on the platelet-derived growth factor receptor β (PDGFRβ) and the downstream pathways including mitogen-activated protein kinases (MAPKs) signalling cascades (Figure 4A). Results showed that PDGF-BB increased the phosphorylation level of PDGFRβ (Figure 4B), ERK1/2 (Figure 4C) and p38MAPK (Figure 4D) in a time-dependent manner, whereas there were no changes in the phosphorylation level of c-Jun N-terminal kinase (JNK) (Figure 4E). When after pretreatment with NBL1, the phosphorylation of PDGFRβ and p38MAPK induced by PDGF-BB were reduced. By contrast, the phosphorylation of ERK1/2 was no change (Figures 5A and 5B). To further investigate p38MAPK involved in the processes of NBL1 suppressing the human PASMCs growth, we used the SB203580, a specific inhibitor of p38MAPK and examined the cyclin D1–CDK4 activity, cell proliferation ratio and the rate of cellular DNA synthesis by co-IP, MTS assay and EDU uptake assay respectively. Our results showed that treatment of SB203580 or NBL1 alone could reduce the cyclin D1–CDK4 activity (Figure 6A upper), p27 phosphorylation and p38MAPK phosphorylation (Figures 6A lower and 6B), the proliferation ratio of human PASMCs (Figure 6C) and EdU uptake value (Figure 6D) respectively. Furthermore, all of the data including the cyclin D1–CDK4 activity (Figure 6A upper), p27 phosphorylation and p38MAPK phosphorylation (Figures 6A lower and 6B), the proliferation ratio of human PASMCs and EdU uptake value were further decreased by SB203580 and NBL1 together (Figures 6C and 6D). These data suggest that PDGFRβ-p38MAPK pathway is impaired in the suppression of human PASMCs proliferation by NBL1, and reduced cyclin D1–CDK4 complex formation and p-p27 are likely the downstream of the blockade of p38MAPK.

## DISCUSSION

Previous studies have showed that the expression of NBL1 was significantly reduced in a wide variety of transformed cells [12] and human tumour specimens [13]. However, Nakamura et al. [14] reported that when added the purified NBL1 to the culture medium, the DNA synthesis of Rous sarcomavirus-transformed 3Y1 cells was inhibited. These observations suggest that NBL1 plays an important role in tumour-suppressive activity. Although our early study have demonstrated that NBL1 is highly expressed in normal rat lung tissue but lowly expressed in a rat model of PAH [8], the biological function of the NBL1 protein remains poorly understand in PASMCs. In the present work, we showed for the first time that NBL1 effectively inhibited PDGF-BB induced human PASMC growth and cellular DNA synthesis by MTS assay and EdU uptake analysis respectively. We also observe the expression of PCNA by western blots because PCNA plays an essential role in the regulation of cell proliferation [15]. Our data show that NBL1 inhibits the PCNA expression in a dose-dependent manner. Our results suggest that NBL1 has a potent suppressive effect on human PASMC proliferation.

PASMC proliferation is central to pulmonary arterial remodelling, which requires PASMCs arrested in G_0_ or G_1_ to enter the cell cycle [16]. It is well known that cell cycle is mediated through many regulatory proteins. Cyclin D1 is generally believed as a positive regulator to regulate cell progression in proliferating cells through activation of cyclin-dependent kinase (CDK) 2, CDK4 or CDK6 [17,18], and the CDKs can be combined with cyclin D1 to form a complex to phosphorylate substrate which is associated with promotion of cell cycle progression [19]. Several evidences are demonstrating the importance of regulation of cyclin D–CDK4 kinase activity in cancer cells [20]. In the present study, we demonstrate that NBL1 reduces cell-cycle positive regulator cyclin D1 and CDK4 in human PASMCs. Moreover, p21 and p27 are the two primary negative regulatory proteins for cell cycle [21]. We find that pretreatment of NBL1 can block the suppressive effect of p27 by PDGF-BB in a dose-dependent manner, but p21 has no change in human PASMCs. It is known that increased levels of negative regulator p27 cause cells to arrest the cell cycle. On the contrary, decreased levels of p27 lead to cell cycle progression [22]. To further confirm the involvement of p27 accumulation in NBL1-induced growth suppression, we use the targeted RNAi-mediated gene knockdown technology. The results show that p27 knockdown impaired the growth suppression induced by NBL1. Phosphorylation of p27 induces to the stabilization of p27 and growth arrest [23]. Previous studies have indicated that p27 is phosphorylated at the terminal threonine, Thr^198^ in human [23,24], so we observe the phosphorylation of p27 by using the anti-phosphorylated p27 at Thr^198^ antibody at 3 h. Our data show that phosphorylated p27 at Thr^198^ is decreased by treatment of NBL1 in human PASMCs, but total p27 does not change. This data is similar with Sakakibara et al.’s research [25]. Therefore, our results suggest that NBL1 suppresses the human PASMCs through inhibition of cyclin D1–CDK4 complex and up-regulation of p27 by decreasing the phosphorylation of p27.

To gain further insights into the molecular mechanisms by which NBL1 reduces cell proliferation in human PASMCs, we examined intracellular signalling pathways. PDGF-BB/PDGFRβ signalling plays a pivotal role in vascular remodelling during cellular and extracellular responses to injury [26], and the PDGFRβ has unique signalling capacity for VSMCs. Activities of PDGF-BB/PDGFRβ signalling are strongly implicated in the pathogenesis of PAH in patients and animal models by initiating and maintaining underlying pulmonary vascular remodelling [27]. Moreover, it has been demonstrated that different signalling pathways mediated by PDGFRβ regulate the proliferative responses to PDGF-BB, particularly with MAPKs signalling pathways [28]. MAPKs are a group of serine/threonine protein kinases comprising three subfamilies: the p42/p44 extracellular signal-regulated kinase (ERK), p38MAPK and JNK [29]. As we known, the classic MAPK pathway is a key component in the transduction of signals leading to growth in many cell types [30]. Previously, several other investigators have shown that ERK and JNK signalling pathways involve in the regulation of cell proliferation in different cell type [31,32]. In our study, phosphorylation of PDGFRβ and its downstream molecules, ERK1/2 and p38MAPK but not JNK are activated by PDGF-BB. However, only the activation of p38MAPK pathway is inhibited by NBL1 pretreatment. We further explore the role of p38MAPK in the regulation of the expression and activity of cell cycle regulatory proteins. Out data show that treatment of the specific p38MAPK inhibitor, SB203580 significantly decrease the cyclin D1–CDK4 complex formation and suppress the phosphorylation of p27. The results reveal a similar effect as treatment with NBL1 alone or together with SB203580. Recently, Ren et al. [26] reported that SB203580 dose dependently blocked PDGF-induced DNA synthesis in PASMCs, which consistent with our data. Our results suggest that the inhibition of p38MAPK activation is likely a major mechanism of growth arrest by NBL1 in PDGF-BB induced proliferation in human PASMCs.

## CONCLUSIONS

**Figure 7:**
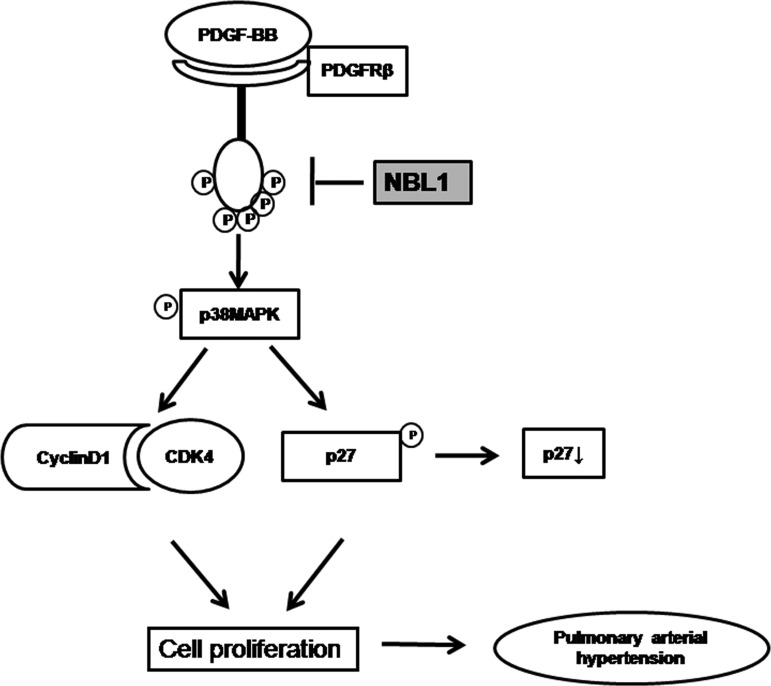
Schematic diagram of proposed NBL1 inhibited PDGF-BB induced human PASMC proliferation in PAH NBL1 inhibits PDGF-BB induced human PASMC proliferation is mediated by decreased cyclin D1–CDK4 activity and up-regulation of p27 by decreasing the phosphorylation of p27 through blocking the PDGFRβ-p38MAPK signalling pathway.

In conclusion, we know from the present studies that NBL1 can inhibit PDGF-BB induced human PASMC proliferation. This process is associated with inhibition of cyclin D1–CDK4 complex and up-regulation of p27 by reducing the phosphorylation of p27 through blockade of PDGFRβ-p38MAPK signalling pathway. These findings are of potential clinical interest and may provide a novel therapeutic target for PAH (Figure 7).

## Abbreviations

CDK2: cyclin-dependent kinase2
CDK4: cyclin-dependent kinase4
CDK6: cyclin-dependent kinase6
EdU: 5-ethynil-2-deoxyuridine
ERK: extracellular signal-regulated kinase
HRP: horseradish peroxidase
IP: immuoprecipitation
JNK: c-Jun N-terminal kinase
NBL1: neuroblastoma suppressor of tumorigenicity 1
PAH: pulmonary arterial hypertension
PASMC: pulmonary artery smooth muscle cell
PCNA: proliferating cell nuclear antigen
PDGF: platelet-derived growth factor
PDGFRβ: platelet-derived growth factor-receptor β
p38MAPK: p38 mitogen-activated protein kinase
RFU: relative fluorescence unit
